# Microbiome Analysis of Carious Lesions in Pre-School Children with Early Childhood Caries and Congenital Heart Disease

**DOI:** 10.3390/microorganisms9091904

**Published:** 2021-09-08

**Authors:** Nelly Schulz-Weidner, Markus Weigel, Filip Turujlija, Kassandra Komma, Jan Philipp Mengel, Maximiliane Amelie Schlenz, Julia Camilla Bulski, Norbert Krämer, Torsten Hain

**Affiliations:** 1 Dental Clinic—Department of Pediatric Dentistry, Justus Liebig University, Schlangenzahl 14, 35392 Giessen, Germany; nelly.schulz-weidner@dentist.med.uni-giessen.de (N.S.-W.); julia.c.bulski@dentist.med.uni-giessen.de (J.C.B.); norbert.kraemer@dentist.med.uni-giessen.de (N.K.); 2 Institute of Medical Microbiology, Justus Liebig University, Schubertstrasse 81, 35392 Giessen, Germany; markus.weigel@mikrobio.med.uni-giessen.de (M.W.); filip.turujlija@bio.uni-giessen.de (F.T.); kassandra.komma@mikrobio.med.uni-giessen.de (K.K.); jan.p.mengel@mikrobio.med.uni-giessen.de (J.P.M.); 3 Dental Clinic—Department of Prosthodontics, Justus Liebig University, Schlangenzahl 14, 35392 Giessen, Germany; maximiliane.a.schlenz@dentist.med.uni-giessen.de; 4 Center for Infection Research (DZIF), Partner Site Giessen-Marburg-Langen, Justus Liebig University, Schubertstrasse 81, 35392 Giessen, Germany

**Keywords:** oral health, pediatric dentistry, early childhood caries, congenital heart disease, microbiota, 16S rRNA gene, dentinal microbiome, next-generation sequencing, dysbiosis

## Abstract

Oral bacteria have been associated with several systemic diseases. Moreover, the abundance of bacteria associated with caries has been found to be higher in patients with congenital heart disease (CHD) than in healthy control groups (HCGs). Therefore, this study aimed to evaluate the dental microbiota in children with CHD compared to a HCG. The aim was to describe and compare the carious microbiome regarding the composition, diversity, and taxonomic patterns in these two groups. Twenty children with CHD and a HCG aged between two and six years participated. All of them were affected by early childhood caries. Microbiome profiling indicated that *Fusobacterium*, *Prevotella, Capnocytophaga*, and *Oribacterium* were more abundant in the CHD group, whereas *Lactobacillus* and *Rothia* were predominant in the HCG. Furthermore, microbiome analysis revealed three distinct clusters for the CHD and HCG samples. In the first cluster, we found mainly the genera *Lactobacillus* and *Coriobacteriaceae*. The second cluster showed a higher relative abundance of the genus *Actinomyces* and a more diverse composition consisting of more genera with a smaller relative lot. The third cluster was characterized by two genera, *Streptococcus* and *Veillonella.* These data can help us to understand the oral microbial community structures involved in caries and endodontic infections of pre-school children in relation to the general health of these high-risk patients.

## 1. Introduction

Early childhood caries (ECC) is the most common childhood disease, affecting more than half of children up to six years of age [1]. Moreover, ECC is often challenging to treat successfully chairside, meaning that affected children must often be treated under general anesthesia [2]. This is a particular problem for children with heart disease who are at high risk of general anesthesia, in whom an increased incidence of caries is described [3]. Studies focusing on the oral microbiota of ECC revealed hundreds of different microbial species in the plaque biofilms of children [4]. Next to caries indicator-microorganism oral commensals such as *Streptococcus mutans* and the genus *Lactobacillus*, members of other genera such as *Bifidobacterium, Actinomyces, Propionibacterium, Veillonella*, and *Scardovia* were identified as potential contributors to the caries microbiota [5,6]. Severe (S)-ECC is an advanced form of dental caries, described to cause acute pain and sepsis [7]. *S. mutans* is predominantly found in S-ECC stages [8], just as *Scardovia wiggsiae* is documented to play a significant role in the advanced stages of caries [9]. Moreover, *Prevotella* has been shown to play a leading role in caries progression in endodontic infections, clinically visible as fistula or abscesses [6].

Oral microbiota are associated with oral diseases such as caries and periodontitis, reflecting systemic conditions [10]. Moreover, the oral microbiome is closely related to systemic diseases such as oral tumors, diabetes, and rheumatoid arthritis, indicating that the oral microbiome can be used as an important marker of early warning for oral and general health [11,12,13,14]. Members of the oral bacterial microbiota are mainly responsible for local and distant-site infections [15]. These infections appear as acute infections such as bacteremia, or even endocarditis [16,17,18,19], with chronic inflammation [20]. They can lead to further problems, especially in this vulnerable group of children with CHD, who are often limited in compliance regarding necessary therapies due to their age.

Several studies showed a relative abundance of bacteria that are often present in the oral cavity—such as *Streptococcus* spp., *Lactobacillus salivarius, Solobacterium moorei*, and *Atoobium parvulum*—to be higher in patients with congenital heart disease (CHD) than in the healthy control group (HCG) [21,22].

A disturbed oral microbiome can have significant effects beyond the oral cavity, especially with regard to the increased risk of infection described above, which imply that patients are not healthy or lack good oral health [23]. Although the exact mechanism of the interaction between infectious diseases and microbiota has not been clarified, the prevention of dysbiosis of the oral microbiota might be a good measure for decreasing the risk of associated infectious complications in diseased children, as shown in a study of children under immunosuppression [24]. This association would necessarily affect children with congenital heart disease.

Limited information is available regarding the oral microbiome associated with CHD, especially in pre-school children. In the present pilot study, 16S rRNA amplicon sequencing was employed to compare the microbiome of the deep dental lesions of CHD pre-school children with a same-aged HCG. The main objectives were to describe and compare the carious microbiome regarding the composition, diversity, and taxonomic patterns in these two groups.

## 2. Materials and Methods

From February 2018 to August 2019, the clinical investigations were conducted at the Department of Pediatric Dentistry, Justus Liebig University, Giessen.

Twenty pre-school children with early childhood caries aged between two and six years participated in the study. Patients with various severities and types of congenital heart defect (CHD), according to the categorization of Warnes et al. [25], could participate if they had undergone a minimum of one heart operation in the past. Others (the HCG) consisted of children with a healthy general condition or without a significant handicap (maximum ASA class I).

The pilot study was conducted following the guidelines of the Declaration of Helsinki and approved by the ethics committee of the Department of Medicine, Justus Liebig University Giessen (ref. no. 186/17; date of approval: 12 February 2018).

### 2.1. Dental Examination and Sampling

Dentists trained in dental measurements examined the children. The principal investigators’ preliminary calibration (interrater reliability; N.S.-W. and J.C.B.) took place in September 2017. The training included a theoretical part explaining the criteria of caries diagnosis and a practical examination exercise on a total of 10 patients of different ages [26]. The intensity of agreement of the study investigator to the reference investigator was very good (κ = 0.83).

A complete dental examination was performed after a supragingival polish, in which carious lesions, and missing and filled teeth (dmf-t) indexes were analyzed [27]. The assessment of caries was purely visual, with classification limited to enamel or dentine caries only. According to WHO criteria, caries levels were determined by the level of cavitation into dentin lesions [28]. Only children affected by dentine carious lesions were recruited. The severity of caries was classified according to the International Association of Paediatric Dentistry (IAPD) criteria [29,30]. According to DAJ (German Association for Youth Dental-Care), the caries risk was evaluated [31]. All examinations were conducted with a plane mouth mirror. Furthermore, concomitant oral findings such as fistula or abscesses were registered.

During therapy, in the course of removal of caries and the treatment need for filling, the carious material was collected by a sterile excavator. The samples were collected in 100 µL sterile ddH_2_O and stored frozen at −80 °C until further analysis.

### 2.2. DNA Extraction and 16S RNA Gene Amplicon Sequencing

Microbial DNA extractions and amplification of the V4 of the 16S rRNA gene were carried out as previously described in Dabrowski et al. [32]. Briefly, genomic DNA was isolated according to the manufacturer’s instructions, following the DNeasy PowerSoil Pro Kit protocol (Qiagen, Hilden, Germany). PCR amplification was performed, using forward and reverse primers [33], to amplify the hyper-variable region V4 of the bacterial 16S rRNA genes. Amplification conditions were used as described previously [34]. Negative tests and PCR controls were performed using only elution buffer from the PowerSoil Pro Kit and nuclease-free water (Qiagen, Hilden, Germany).

PCR products were purified using AMPure XP DNA beads (Beckman Coulter, Krefeld, Germany). The quality of the libraries was assessed using a Qubit Fluorometer 2.0 (Thermo Fisher Scientific, Waltham, MA, USA) and the 2100 Bioanalyzer system (Agilent Technologies, Frankfurt, Germany). Purified amplicons were quantified by PicoGreen dsDNA assay (Thermo Fisher Scientific, Waltham, MA, USA), according to the manufacturer’s instructions, and samples were diluted, pooled, and spiked with 15% PhiX. Finally, libraries were loaded for paired-end sequencing on the Illumina MiSeq platform using v2 chemistry (2 × 250 cycles).

### 2.3. Statistical Analysis/Bioinformatics

Microbiome analysis was executed using Mothur [35]. Paired-end reads were joined, primer regions removed and filtered for the expected amplicon length of 253 nt ± 10 nt, excluding sequences that contained ambiguous nucleotides. Joined paired-end reads were aligned to the SILVA ribosomal RNA gene database [36], trimmed to contain only the hypervariable region V4 and clustered with a similarity threshold of 97%. After chimera removal using VSEARCH [37], operative taxonomic units (OTUs) were obtained and classified against the SILVA ribosomal RNA gene database. For further analysis, we subsampled all samples to 3000 reads. Mothur, rarefaction curves, principal coordinate analysis (PCoA) of the Bray–Curtis dissimilarity, linear discriminant analysis (LDA) effect size (LEfSe) [38], analysis of molecular variance (AMOVA) and homogeneity of molecular variance (HOMOVA) were created/executed. OTUs that we could not classify to the genus level were further analyzed by BLASTn [39] against the 16S ribosomal RNA database from the NCBI RefSeq Targeted Loci Project [40]. Results with a *p*-value < 0.05 were considered significant.

## 3. Results

Twenty pre-school children participated in the study. Eleven fulfilled the criteria for congenital heart disease (CHD), of which two presented a mild congenital heart defect and nine severe CHD. Nine children represented the healthy control group (HCG). The two groups were almost balanced by gender and carious status (dmf-t index). No significant difference (*p* > 0.05) could be observed between CHD and HCG regarding the dmf-t index (Supplemental Materials Table S1).

**Figure 1:**
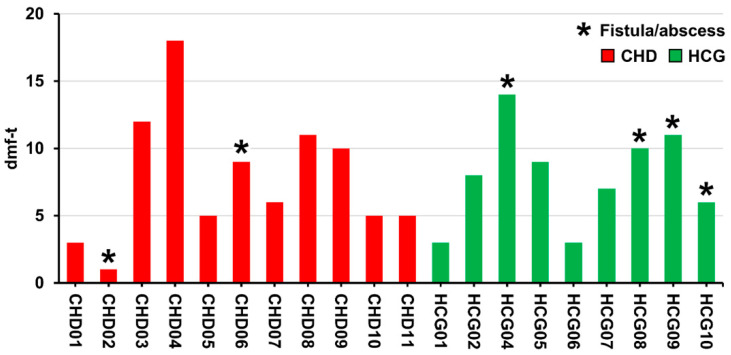
Bar chart of dmf-t values for each patient with congenital heart disease (CHD) or healthy control group (HCG).

**Figure 2:**
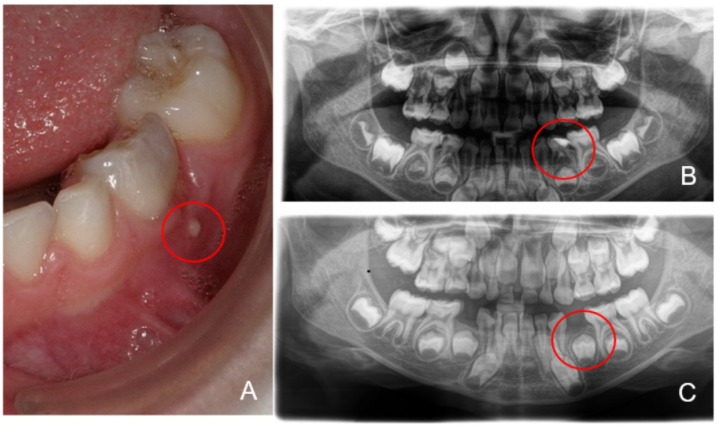
Patient CHD06 with severe early childhood caries (S-ECC) and fistula (marked by the red circle) of decayed tooth 74 (**A**), radiographic showing parodontitis periapicalis chronica as a result of endodontic infection with the need for extraction of tooth 74 (marked by the red circle; **B**) and the post-therapy recall after extraction tooth 74 after three years (marked by the red circle; **C**).

All the children presented a high caries risk with dentin carious lesions. According to the IAPD, 13 of the children showed S-ECC [29,30] (Figure 1). Six patients showed clinical signs of inflammation, revealing fistula/abscess (Figure 1), including two children with CHD. Figure 2 shows patient CHD06 with S-ECC (dmf-t = 9) and fistula in the region of the lower first primary molar (FDI #74) as an expression of endodontic infection after profound carious destruction with the need for extraction to avoid complications.

**Figure 3:**
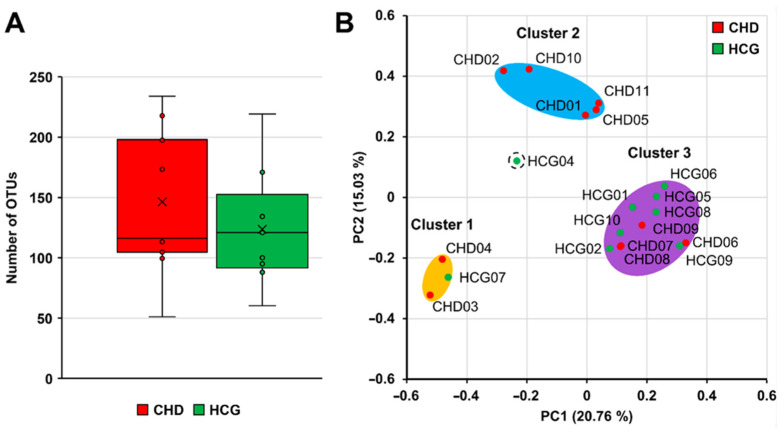
Boxplot diagram based on the number of OTUs observed at a sampling depth of 3000 reads for the children with CHD and a HCG (**A**). PCoA of Bray–Curtis dissimilarity for the CHD and HCG samples (**B**).

The boxplot diagram based on the number of observed OTUs at the sampling depth for the two different patient groups shows a similar mean and median. While a greater variation was observed in the CHD group, we found no statistically significant difference between children with CHD and those in the HCG (Figure 3A).

PCoA of the Bray–Curtis dissimilarity gave three distinct groups for the CHD and HCG samples. The first cluster (CHD03, CHD04, HCG07) and the third cluster (CHD06, CHD07, CHD08, CHD09, HCG01, HCG02, HCG05, HCG06, HCG08, HCG09, HCG10) are a combination of CHD and HCG samples, while the second cluster (CHD01, CHD02, CHD05, CHD10, CHD11) contains only samples from children with CHD. Sample HCG04 was not assigned to any of the three clusters. We neither observed a significantly different centroid with AMOVA nor did HOMOVA show a significant difference in the variation between the CHD and HCG samples overall. Samples split into the three clusters showed a significantly different centroid (Figure 3B).

On the highest classification level, we found a total of 170 distinct taxa; 62 of those had a relative abundance of at least 1% in any sample. Overall, we found comparable amounts of *Lactobacillus*, *Neisseria*, and *Streptococcus* in the CHD and HCG samples. The mean of *Veillonella* was twice as much in the HCG than for those with CHD. Additionally, *Olsenella* and *Rothia* were increased in the HCG. In contrast, the average for *Actinomyces* in the CHD group was twofold higher than in the HCG. Furthermore, *Fusobacterium* was more abundant in the CHD samples.

In the first cluster, we found mainly *Lactobacillus*, and in sample CHD04, we identified *Olsenella* as the most likely genus. Two samples (CHD01 and CHD05) of the second cluster showed a higher relative abundance of *Actinomyces*. Otherwise, we noticed a more diverse composition consisting of a more significant number of genera with a smaller relative lot. Of those, *Corynebacterium*, *Fusobacterium*, *Leptotrichia*, *Prevotella*, *Selenomonas*, and *Veillonellaceae* were the most noteworthy. The most abundant OTU for the family *Veillonellaceae* in the second cluster was identified by BLASTn as different *Selenomonas* species. The third cluster was characterized by *Streptococcus* and *Veillonella*.

**Figure 4:**
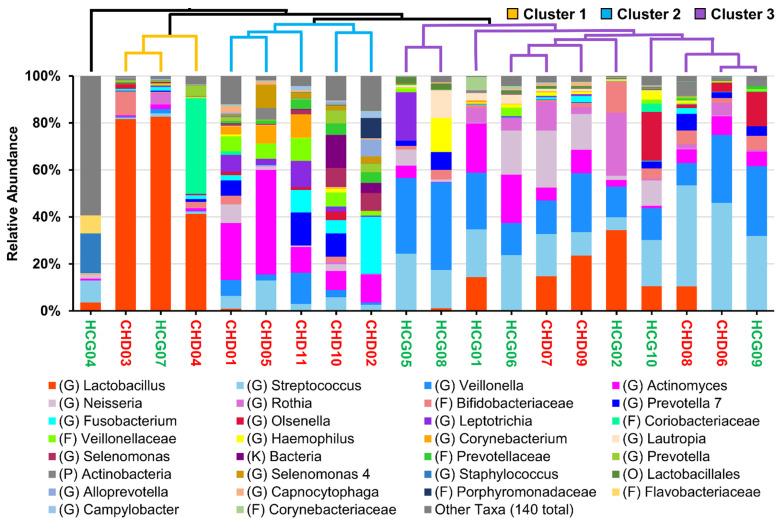
Cumulative bar charts showing the relative abundance of the top 30 taxa by the mean abundance of their highest classification level. Taxa not in the top 30 are summarized as ‘other Taxa’. The dendrogram on the top shows a tree based on the Bray–Curtis dissimilarity using the UPGMA algorithm [(G): genus; (F): family; (O): order; (P): phylum; (K): kingdom]. Sample name is given in red for CHD group and in green for HCG group.

Furthermore, we found *Actinomyces*, *Neisseria*, *Lactobacillus*, *Olsenella*, and *Rothia* to be major genera in at least half of the samples. *Bifidobacteriaceae* further identified as *Parascardovia*, *Haemophilus*, and *Leptotrichia* was detected each in at least one instance to be above 10%. The composition of sample HCG04 consisted primarily of *Flavobacteriaceae*, *Staphylococcus*, and *Streptococcus*. Additionally, we found *Anaerococcus* and *Bacillus* to make up a major group under the other genera (Figure 4).

**Figure 5:**
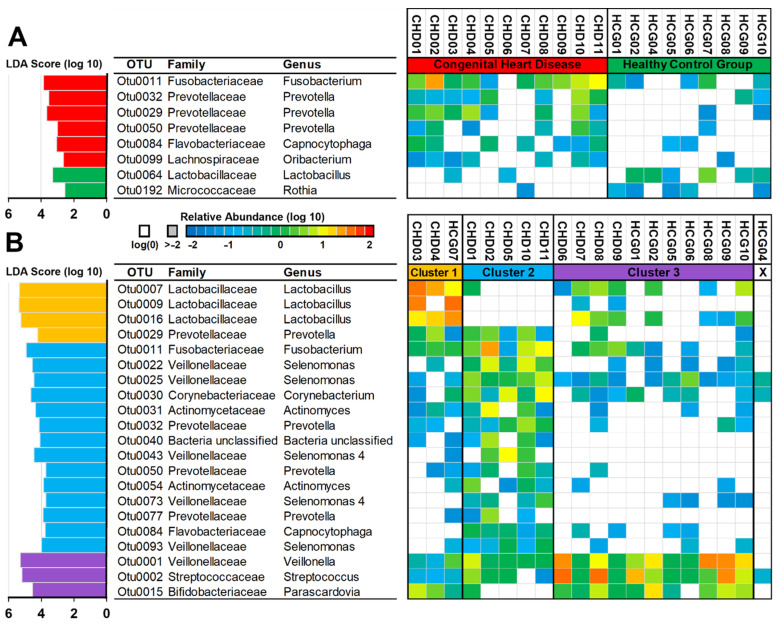
LEfSe for CHD vs. HCG (**A**) and the three distinct clusters observed in the PCoA of Bray–Curtis (**B**). LDA score for significantly different OTUs between the three clusters (left), taxonomic classification (center), and heatmap showing the log-10 transformed relative abundance for each OTU (right).

To further characterize the unique composition of the CHD and HCG groups, and the three clusters on an OTU level, we utilized LEfSe (Figure 5). For the CHD group, we found primarily *Fusobacterium* and additionally *Prevotella*, *Capnocytophaga*, and *Oribacterium* OTUs with a significantly higher abundance. HCG samples showed a single OTU for *Lactobacillus* and *Rothia* as significantly discriminative features.

Aligning with our results on the genus level, we found *Lactobacillus* to be the primary characteristic genera of the first cluster. The number of significant results for the second cluster was the largest. This coincided with the more diverse composition we observed. We found multiple OTUs for *Actinomyces*, *Prevotella*, and *Selenomonas* and single OTUs for *Fusobacterium*, *Corynebacterium*, and *Capnocytophaga* as significant differentially expressed. For the third cluster, we could confirm the dominance of *Veillonella* and *Streptococcus*.

## 4. Discussion

Even though other groups have characterized the microbiome in patients, to our knowledge, this is the first study that examines the carious microbiome of pre-school children with congenital heart disease (CHD) compared to a healthy control group (HCG) using next-generation sequencing. With the 16S rRNA amplicon sequencing technique, we determined the microbiota profile of dentinal lesions of CHD and HCG children and determined several species associated with caries, as described in the literature. These included the genera *Streptococcus, Lactobacillus, Prevotella*, *Veillonella, Bifidobacterium, Fusobacterium, Selenmonas, Corynebacterium, Actinomyces, Selemonas*, and *Capnocytophaga,* which have been assigned to the caries genesis process [41].

Comparison between CHD and HCG individuals showed that *Fusobacterium*, *Prevotella, Capnocytophaga*, and *Oribacterium* were more abundant in the CHD group, whereas in the HCG, *Lactobacillus* and *Rothia* were the discriminative features. *Fusobacteira* played an essential role in caries formation [42]. The fact that the species was found in the majority of children in the particularly vulnerable group of CHD children is of considerable interest because these microbes can enter the general circulation and cause bacteremia. The result can be harmful systemic effects that can promote diseases, as oral bacteria are responsible for many infections and thus for further systemic problematic concomitant conditions for affected children. We further investigated by PCoA of Bray–Curtis dissimilarity how individuals of both groups clustered among each other. Regarding cluster 1, there was a high monomicrobial ratio of *Lactobacillus*, whereas the CHD cluster (cluster 2) was composed of *Fusobacterium*, *Veillonella*, *Corynebacterium*, *Actinomycetes*, *Prevotella*, and *Flavobacterium.* Cluster 2 showed the highest diversity in potential contributors to ECC [43]. In cluster 3, the genera of *Veillonella*, *Streptococcus*, and *Bifidobacterium* were observed.

According to the literature, *Lactobacillus* was found at low levels in endodontic infections with deep carious lesions [44], similar to those that were present in these subjects in cluster 1. It has also been suggested that altered *Lactobacillus* abundance may be due to the shift from cariogenic microbiota to a bacterial composition that stimulates progression into pulpal tissue, causing infection [45].

Furthermore, we indicated the genus *Olsenella*, which belongs to the family of *Coriobacteriaceae*, in four of eleven samples of the individuals in cluster 3. NCBI rRNA database BLAST analysis of V4 sequencing reads identified *Olsenella profusa* as the best matching species. The genus *Olsenella* is well known to cause endodontic infections in humans [46,47], which might be the causal agent for the abscess of CHD06 as depicted in Figure 2. Although we were unable to demonstrate clinical signs of infection (fistula, abscess) in the majority of the subjects, we nonetheless cannot rule out the possibility that pulpal necrosis with incipient endodontic infection had already occurred regarding the depth of the dentinal lesion.

Moreover, the most abundant species, which is described to be associated with ECC, *S. mutans,* was detected and confirmed in best hit BLAST analysis using the NCBI rRNA database [48,49,50]. *Veillonella* spp., described to contribute to caries progress [51] and *Bifidobacterium*, significantly associated with S-ECC [52], were identified. These findings correspond with our clinical findings, which showed high dmf-t values (S-ECC) in all patients and clinical inflammation in five of the patients (CHD06, HCG10, HCG08, CHD02, and HCG04). Moreover, these two genera, as known early colonizers of tooth surfaces, interact to form dental plaques: *Streptococcus* produces a preferred fermentation product for *Veillonella* [53,54,55,56,57].

We showed that bacterial diversity and composition differed in all three clusters. These results suggest that there might be an impact of different health aspects on the oral microbiome. In our study, the oral microbiota of cluster 1 and cluster 3 had fewer diverse results compared to cluster 2 (CHD group). In our research, we could detect a high diversity of *Veillonella* in our clustered group of CHD children. In view of these results, it can be assumed that some aspect of CHD could influence the composition of the oral microbiome. Also, in view of other studies in which species from caries, plaque and saliva were examined, it can be assumed that the results for the carious lesion are transferable to the oral cavity as a whole. Furthermore, oral colonization appears to have systemic influences [58].

Dental disease has been associated with an increased risk of infection, suggesting that bacteria from the oral cavity may contribute to the development of inflammation [20]. With regard to poor oral health’s potential systemic consequences, these should be considered with regard to causes of infection such as bacteremia. The harmful systemic effects that result could thus be responsible for further systemically problematic concomitant conditions in children. Therefore, prevention of dysbiosis of the oral microbiome could be a promising measure to reduce the risk of infectious complications in sick children, especially in children with congenital heart disease [24,59]. Dental disease has been associated with increased risk of infection, suggesting that bacteria from the oral cavity may contribute to the development of inflammation.

Some limitations should be considered for this study. At the clinical level, caries diagnosis was based on clinical assessment of the dentinal lesion. Thereby, the principal examiner (N.S.-W. and J.C.B.) underwent calibration prior to clinical data collection with intra-examiner calibration (with 10 volunteers not recruited to the study) to verify the diagnosis. Accordingly, a strong bias could be prevented regarding diagnosis. Furthermore, the sizes of both cohorts were limited; further study warrants extension in group size to confirm these preliminary results. In addition to reduce possible amplification bias, triplicate PCR reactions could be employed. However, as the small, investigated population of patients exhibited differences, it can be suggested that the number of individuals might have been sufficient in our pilot study. Since the literature on children is limited with regard to the microbiome and heart disease, we referred to the literature on adults, as the results could represent a possible trend for children as well.

Our results indicate that dental health seems to play a role in overall health; this warrants further examination with a larger number of participants in both groups of those with heart disease and healthy pre-school children.

## 5. Conclusions

Within the limitation of this being a preliminary study, it can be concluded that the dentinal microbiome differs between CHD children and a HCG. Furthermore, we observed three distinct groups with different microbiome profiles, which indicates the need for a more individualized risk assessment for the CHD individuals. Thus, oral health appears to be of immense importance, especially regarding vulnerable groups, and seems to play an important role with regard to possible complications. These data can help us to understand oral microbial community structures involved in caries and endodontic infections in pre-school children regarding the general health of these high-risk patients.

## Data Availability

Microbiome sequencing data have been submitted to the NCBI Short Read Archive repository under the BioProject accession number PRJNA731066 (https://www.ncbi.nlm.nih.gov/sra/PRJNA731066, accessed on 6 September 2021).

## Supplementary Materials

The following are available online at https://www.mdpi.com/article/10.3390/microorganisms9091904/s1, Table S1: Metadata for each patient with congenital heart disease (CHD) and healthy control group (HCG) indicating sex (f = female, m = male), age in month, classification of heart disease (when applicable), dmf-t value and presence of fistula/abscess.
