# Supplementary material for: Microbiome Analysis of Carious Lesions in Pre-School Children with Early Childhood Caries and Congenital Heart Disease
Source: Microorganisms. 2021 Sep 8;9(9):1904. doi: 10.3390/microorganisms9091904 (PMC8469307; doi:10.3390/microorganisms9091904)
Supplement: Supplementary file 1 [file microorganisms-09-01904-s001.zip › microorganisms-1268612-supplementary.pdf]

Table S1. Metadata for each patient with congenital heart disease (CHD) or healthy control group (HCG) indicating sex (f = female, m = male), age in month; dmf-t value and presence of fistula/abscess, classification of heart disease, heart surgery and medication (where applicable), (n.a. = not applicable).

Patient	Sex (m/f)	Age (in months)	dmf-t	fistula/ abscess	CHD (Warnes Classification)	Heart surgery	medication
CHD 01	f	62	3	-	Hypoplastic left heart syndrome (3)	-	
CHD 02	m	36	1	+	Tricuspid atresia (3)	Fontan-Operation, Glenn-Procedure	Acetylsalicylic acid
CHD 03	m	67	12	-	Pulmonary insufficiency/ interrupted aortic arch (3)	Fontan-Operation, Comprehensive stage 2 procedure	Acetylsalicylic acid, ACE inhibitor, Beta-Blocker
CHD 04	m	24	18	-	Pulmonary atresia (3)	-	Diuretics
CHD 05	f	60	5	-	Hypoplastic left heart syndrome/ VSD (3)	Glenn anastomoses, Norwood operation	Acetylsalicylic acid
CHD 06	f	36	9	+	Mital valve insufficiency (3)	LIMA-LAD Bypass	-
CHD 07	m	36	6	-	Tetralogy of Fallot (3)	Conduit replacement, VSD -Closure	Beta-Blocker, Diuretics
CHD 08	f	62	11	-	VSD, Truncus arteriosus communis (3)	VSD-Closure, Aortic reconstruction	-
CHD 09	m	62	10	-	ASD (1)	-	Acetylsalicylic acid
CHD 10	m	61	5	-	ASD (1)	-	-
CHD 11	m	63	5	-	Patent foramen ovale (1)	n.a.	n.a.
HCG 01	f	64	3	-	n.a.	n.a.	n.a.
HCG 02	m	62	8	-	n.a.	n.a.	n.a.
HCG 04	f	69	14	+	n.a.	n.a.	n.a.
HCG 05	m	64	9	-	n.a.	n.a.	n.a.
HCG 06	m	63	3	-	n.a.	n.a.	n.a.
HCG 07	m	67	7	-	n.a.	n.a.	n.a.
HCG 08	f	24	10	+	n.a.	n.a.	n.a.

HCG 09	f	62	11	+	n.a.	n.a.	n.a.
HCG 10	f	62	6	+	n.a.	n.a.	n.a.
